# Comparative Metagenomic Studies Reveal Different Evolutionary Directions of Synthetic Indoor Microbial Communities Under Different Nutritional Conditions

**DOI:** 10.3390/ijms27104238

**Published:** 2026-05-10

**Authors:** Xinyi Zhang, Lin Cai, Yukun Bai, Fang Peng

**Affiliations:** China Center for Type Culture Collection, College of Life Sciences, Wuhan University, Wuhan 430072, China; 2023302041036@whu.edu.cn (X.Z.); cailin@whu.edu.cn (L.C.); yukunbai2023@163.com (Y.B.)

**Keywords:** indoor microbial communities, metagenomics, metatranscriptomics, synthetic microbial communities, microbial ecology, human health

## Abstract

The relationship between microorganisms and human health is inseparable. In today’s increasingly urbanized world, the relationship between indoor microbial communities and human health is particularly close. Studies have shown that the composition of indoor microbial communities is influenced by various factors, including temperature, humidity, and nutrient conditions. However, research on how to alter indoor microbial community structures by adjusting nutrient components to improve human health is still limited. In this work, we constructed artificial microbial communities composed of common indoor microorganisms, and analyzed the species composition, metabolic capabilities, antibiotic resistance, and virulence of the microbial communities before and after cultivation using metagenomic sequencing technologies and metatranscriptomic sequencing technologies. We then assessed their community characteristics and evolutionary direction under different nutrient conditions. Overall, when the nutrient conditions were altered and reduced, the evolutionary direction of indoor microbial communities changed significantly. Specifically, this evolutionary direction was manifested in a taxonomic succession of community composition, with marked shifts in the relative abundances of constituent species, as well as in a significant alteration of the community-level metabolic functions. In-depth research in this field can help improve the composition of indoor microbial communities, thereby benefiting human health and public health construction in urbanized environments.

## 1. Introduction

In recent years, with the development of sequencing technologies and the reduction in costs, the analysis of microbial communities has grown explosively [1]. However, despite the rapid increase in available metagenomic datasets, natural community analysis still faces significant limitations in answering fundamental ecological and evolutionary questions surrounding natural microbial communities [2]. Consequently, laboratory models for the investigation of microbial ecology have been established. Synthetic microbial communities have simpler compositions, are easier to study, and are more reproducible than natural ecosystems that are complex and hard to control [1,3]. So far, synthetic biological communities have been used to model how microbes interact in different ecological settings, like the gut, marine ecosystems, and systems where plants and microbes interact [4,5,6,7].

Indoor microbiomes have a very close relationship with human health compared to other microbial communities in different ecological environments [8]. An unbalanced indoor microbiome can cause a number of diseases, which is bad for human health [9,10]. Microbes usually have a hard time growing in dry, nutrient-poor indoor environments, but they will still build up and grow into communities as long as the conditions are right [11,12]. Different environmental factors will cause certain species to become dominant. There are many things that affect indoor microbiomes, but human activity is the most important one [13]. Nutritional conditions are one of the most important things that affect how microbes evolve [14]. The microbial patterns seen on different surfaces in different indoor spaces are closely linked to the nutritional conditions [15,16]. Recent research indicates that employing innovative substrates to obstruct microbial organic carbon assimilation, or incorporating genetically modified probiotic spores into substrate materials, can effectively eradicate conditionally pathogenic bacteria within edifices [17,18]. Despite these studies, the succession and competition of indoor microbes under different nutritional conditions remain unclear, and the ecological mechanisms at the molecular level still need to be elucidated. In-depth research in this field will help to increase our understanding of indoor microbiomes and improve public health in an increasingly urbanized world.

At present, common approaches for studying indoor microbiomes include 16S rRNA sequencing, metagenomic analysis, and emerging innovations such as synthetic DNA–based techniques [19,20,21,22]. Although these approaches can accurately capture the composition and evolutionary characteristics of indoor microbial communities, several limitations remain—for example, they often cannot quantify the absolute abundance of pathogens; when environmental factors are complex, highly variable, and uncontrollable, it is also difficult to characterize community commonalities and explore the core succession patterns [13,14,23]. These challenges are attributable, in part, to methodological constraints. Thus far, indoor microbiome research has primarily relied on sample collection followed by sequencing and statistical analyses, with limited use of laboratory-constructed synthetic models. To address this gap, we introduced an artificial microbial community. In contrast to studies that survey natural communities, we maintained the artificial community over extended cultivation and applied multi-omics strategies to investigate how its evolutionary trajectory changes across conditions.

In this study, building on prior work describing the species composition of indoor microbiota, we constructed an artificial microbial community representative of common indoor species and cultured the mixed community in media spanning different nutrient concentrations [11,19,24]. We then integrated whole-genome sequencing, metagenomic sequencing, and metatranscriptomic sequencing to obtain robust profiles of individual bacterial populations before and after cultivation. The stable laboratory culture environment circumvents complex environmental variables, thereby enhancing the stability and reproducibility of the experimental conclusions. We sought to address the following three questions: (1) How does the direction of succession in indoor microbial communities differ under distinct nutritional conditions? (2) At the molecular level, what pathogenic changes can occur in microorganisms due to changes in environmental nutritional conditions? (3) Under different nutritional conditions, how does the relationship between indoor microbial communities and human health change? By combining bioinformatics analyses with multi-omics datasets, this study aims not only to elucidate how nutritional conditions shape indoor community succession but also to dissect the molecular mechanisms that drive community shifts, with important implications for improving urban ecological environments and advancing public health.

## 2. Results

### 2.1. Variations in Medium Concentration Markedly Shifted Community Successional Trajectories

**Figure 1:**
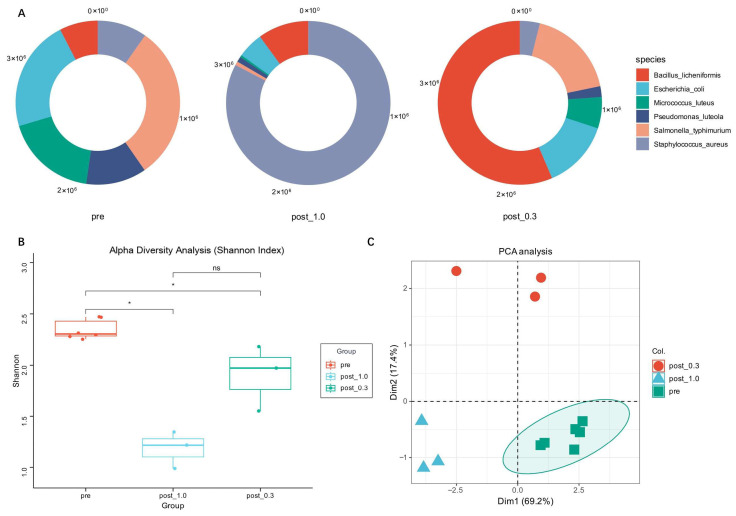
Analysis of species abundance and diversity in synthetic microbial communities. (**A**) Species abundance analysis. (**B**) Alpha diversity analysis was conducted using the Shannon index. One-way ANOVA, * *p* < 0.05. (**C**) Principal Component Analysis. Green ellipse indicate the 95% confidence region.

From common indoor microorganisms, we selected six species that are representative in both taxonomy and indoor distribution [8] and mixed their pure cultures to assemble a synthetic microbial community (Table 1), designated as pre [25,26]. We performed metagenomic and metatranscriptomic sequencing on the synthetic community and then split it into two groups that were cultivated in 1.0× and 0.3× LB medium, designated as post 1.0 and post 0.3, with three replicate samples per group. After two months of co-cultivation, high-throughput sequencing was performed again. Based on the gene sequences obtained from sequencing and their relative abundances, we performed NR species annotation on the microbial communities of each group to analyze species abundance and the proportion of each species within the community (Figure 1A).

NR annotation results showed that compared to before cultivation, the species abundance of the bacterial community changed significantly after cultivation. It also exhibited different successional patterns in 1.0× LB medium and 0.3× LB medium. In both post-cultivation groups, dominant species emerged that accounted for more than half of the total abundance, specifically *Staphylococcus aureus* and *Bacillus licheniformis*. In addition to the absolute dominant species, *Bacillus licheniformis* exhibited stronger competitive fitness in the 1.0× LB medium, while *Escherichia coli* and *Salmonella typhimurium* showed stronger competitive abilities in the 0.3× LB medium, maintaining a certain population size. The remaining strains accounted for very low proportions in the community and occupied non-dominant positions.

Subsequently, we measured community diversity using the Shannon index (Figure 1B). The community complexity in both post-cultivation groups decreased significantly compared to the initial state, while no significant differences were found between the experimental groups. The results indicate that the species structure of the indoor microbial community, formed by representative species, underwent significant changes under different nutrient concentrations, but the diversity approached convergence. Consistent with these results, the Principal Component Analysis (PCA) clustering plot (Figure 1C) shows the changes in species diversity between the experimental groups relative to the initial state, meaning that the experimental groups cluster far from the control group. Dimensions 1 and 2 described most of the variation, indicating that the proliferation of dominant microbial groups was the main factor causing changes in community structure.

### 2.2. The Growth Rate of Strains in Synthetic Communities Differs from That in Individual Cultures

**Figure 2:**
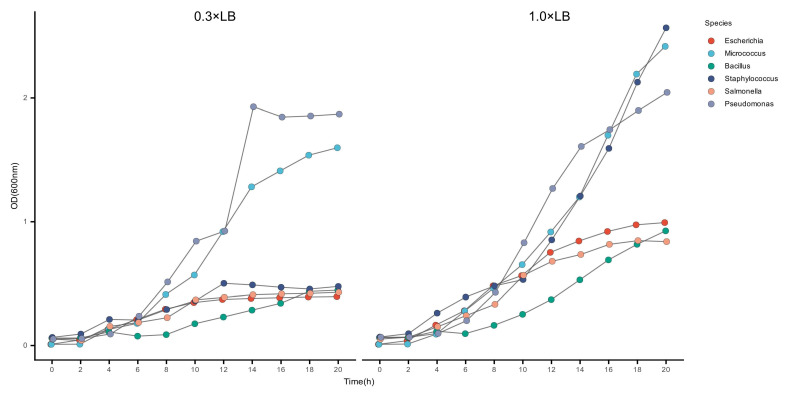
Growth curve measurement of strains constituting the synthetic community. The left side is cultured in 0.3× LB medium, and the right side is cultured in 1.0× LB medium.

After conducting preliminary analysis, we cultured the strains used in the experiment separately and measured their growth curves (Figure 2). It can be observed that bacteria in the 1.0× LB medium had a higher growth rate and biomass during the stable growth phase compared to strains in the 0.3× LB medium. It can also be observed that in both groups, *Pseudomonas luteola* and *Micrococcus luteus* had relatively high growth rates, while *Staphylococcus aureus* grew much faster in 1.0× medium than in 0.3× medium. This explains why *Staphylococcus aureus* occupies the greatest advantage in the artificial community cultured in 1.0× LB medium, demonstrating its high growth potential under nutrient-rich conditions. Additionally, we found that *Pseudomonas lutrola* and *Micrococcus luteus*, which grow faster when cultured alone, performed poorly in mixed culture, whereas *Bacillus licheniformis*, which grows slower when cultured alone, had certain advantages in mixed culture. This indicates that in artificial communities, competition and interaction mechanisms among populations affect the growth rates of strains. These mechanisms may further influence the succession direction of the community and shape the community structure.

### 2.3. Significant Differences in Nutrient Conditions Markedly Alter Community Functions

**Figure 3:**
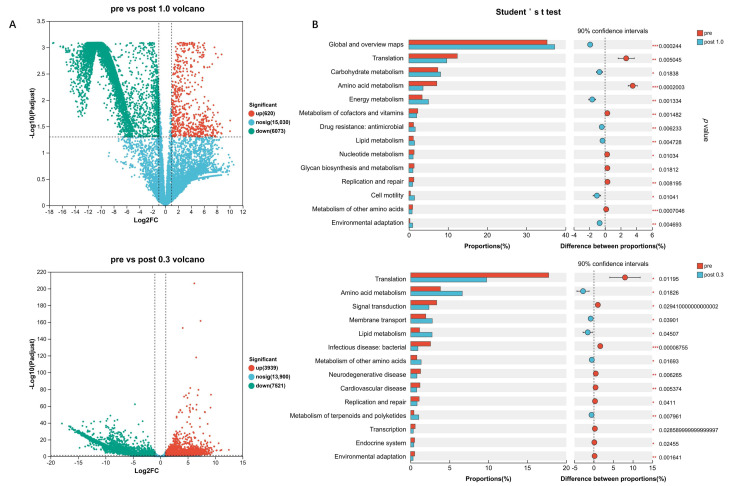
Transcript abundance and functional analysis of synthetic microbial communities. (**A**) Differential gene expression analysis, using TPM as the expression level metric, *p* < 0.05 and |log2FC| ≥ 1. (**B**) Intergroup functional difference test, using TPM as the expression level metric, annotated with KEGG Pathway Level 2, two-sample t-test. 0.01 < *p* ≤ 0.05 *, 0.001 < *p* ≤ 0.01 **, *p* ≤ 0.001 ***.

In order to further clarify the functional changes and trends of the community, we used transcripts per million (TPM) as the abundance indicator, performed differential expression analysis on the metatranscriptome, and screened genes with expression fold change (FC) greater than twofold. We found that, compared with the control group, in the two post-cultivation communities, the number of genes with downregulated transcript abundance was greater than that of upregulated genes (Figure 3A). This indicates that the microbial community is facing strong selection pressure at this time, which is consistent with the phenomenon in the metagenomic species-abundance analysis that a single species occupies an absolute advantage in the community.

Next, we used the KEGG database to perform functional annotation on the metatranscriptomic sequence data and conducted inter-group difference tests between the control group and the treatment groups at the pathway level 2 to analyze significant functional differences before and after co-cultivation of the artificial community (Figure 3B). This analysis showed that, in the 1.0× LB medium group, the expression levels of genes related to pathways such as translation, amino acid metabolism, polypeptide metabolism, cofactor and microbial metabolism, polysaccharide synthesis and metabolism, and replication and repair decreased significantly (*p* < 0.05). Meanwhile, the expression levels of genes related to pathways such as the global and overview maps, carbohydrate metabolism, energy metabolism, and drug resistance—antimicrobial, lipid metabolism, motility, and organismal environmental adaptation—increased significantly (*p* < 0.05). Analysis results indicated that the energy metabolism level of the community increased; the metabolic level of synthetic substances decreased; and the dispersal ability, adaptability, and drug resistance of the community were enhanced to some extent. The competitive ability of the community increased, and it was easier to spread.

In the 0.3× LB medium group, the expression levels of pathway genes related to translation, signal transduction, bacterial infectious diseases, neurodegenerative diseases, cardiovascular diseases, replication and repair, transcription, endocrine system, and organismal environmental adaptation decreased significantly (*p* < 0.05), while the expression levels of genes related to amino acid metabolism, membrane transport, lipid metabolism, and the metabolism of terpenoids and polyketide compounds increased significantly (*p* < 0.05).

The analysis results show that the pathogenicity of the community has decreased, while multiple metabolic capabilities have increased. They also suggest an increase in the synthesis of secondary metabolites in the group. Interestingly, cross-comparison of the two treatment groups revealed that the community under the higher nutrient concentration exhibited greater dispersal ability and higher virulence-related transcription, suggesting that this community may possess stronger virulence in natural environments. In contrast, the community under the lower nutrient concentration displayed more active metabolic processes and lower virulence-associated transcript levels. We believe this difference is related to the competitive interactions between the dominant species in the two communities—*Staphylococcus aureus* and *Bacillus licheniformis*.

### 2.4. Obvious Shifts in the Competition Relationship Between Antimicrobial Substances and Bacterial Drug Resistance Exist Along a Nutrient Concentration Gradient

We performed whole-genome sequencing and de novo assembly for two major strains in the synthetic microbial community: *Bacillus licheniformis* CCTCC AB 91061 and *Staphylococcus aureus* CCTCC AB 91093. Then, through genome annotation, we revealed their protein-coding genes, tRNA genes, and rRNA genes (Table 2). Subsequently, through reference-genome annotation, we revealed genes in the genome of strain CCTCC AB 91061 that are related to antimicrobial active substances (Table S1), and the obtained gene annotations included nonribosomal peptide synthetases (NRPSs), the antimicrobial peptide LCI, and lactococcin 972. Among them, NRPSs corresponded to the biosynthetic pathways of lichenysin and bacitracin; lichenysin belongs to lipopeptides and has been confirmed to inhibit the adhesion of staphylococci and pseudomonads and biofilm formation, whereas bacitracin has broad-spectrum antimicrobial activity against Gram-positive bacteria [27]. The antimicrobial peptide LCI is a cationic antimicrobial peptide (AMP) with strong antimicrobial activity and broad-spectrum antimicrobial activity, and lactococcin 972 can inhibit bacterial septum formation, thereby inhibiting bacterial binary fission [28,29].

**Figure 4:**
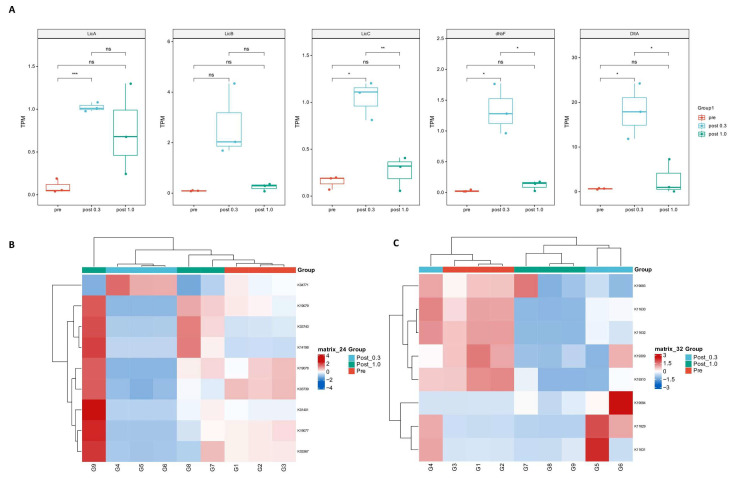
Analysis of antimicrobial peptide and antibiotic resistance gene expression based on metatranscriptomics. (**A**) NRPSs Gene expression analysis, using TPM as the expression level metric and One-Way ANOVA, 0.01 < *p* ≤ 0.05 *, 0.001 < *p* ≤ 0.01 **, *p* ≤ 0.001 ***. (**B**) Heatmap analysis of cationic antimicrobial peptide resistance gene expression, using transcript reads as a measure of expression. (**C**) Heatmap analysis of bacitracin resistance genes and efflux gene expression levels, using transcript reads as a measure of expression.

We used metatranscriptomic sequencing data to analyze the differences in antimicrobial substance expression levels under different nutritional conditions. Due to the sequencing precision, it was difficult to analyze genes with small transcript numbers and short lengths, such as peptide LCI and lactococcin 972. Therefore, we conducted abundance analysis using NRPSs-related genes as representatives (Figure 4A). The data shows that, compared to pre-cultivation, the transcript abundance in both post-cultivation 0.3 and 1.0 groups increased. In the 0.3 group, gene expression was significantly upregulated in most categories (*p* < 0.05), and there were also significant differences when compared to the 1.0 group (*p* < 0.05). This indicates that, in the 0.3 group, the expression of antimicrobial substances in *Bacillus licheniformis* increased significantly compared to the control group, whereas no significant difference was observed in the 1.0 group.

Subsequently, we annotated the whole genome of *Staphylococcus aureus* CCTCC AB 91093 using the KEGG database, selected key resistance genes related to cationic antimicrobial peptides and bacitracin, and performed a heatmap analysis using the KEGG annotation from the metatranscriptome. We found that, in the cationic antimicrobial peptide resistance heatmap, the transcript numbers of most genes in the 0.3 group decreased, while in the 1.0 group, transcript numbers increased (Figure 4B). This indicates that *Staphylococcus aureus* in the synthetic microbial community has stronger resistance under nutrient-rich conditions, which partly explains why this strain dominates the community in the 1.0 group. Interestingly, for the bacitracin resistance genes, transcript changes showed different trends. Among them, the transcripts of some genes were unchanged or decreased in the 1.0 group, while some genes were upregulated in the 0.3 group (Figure 4C). We annotated the genome of CCTCC AB 91061 and found that these pathways are related to the secretion of bacitracin and are homologous to resistance genes, which explains this phenomenon. In summary, through whole-genome and metatranscriptomic analysis, we revealed the interplay between antimicrobial substances and bacterial resistance during cultivation, and this competitive mechanism, which fluctuates under different nutritional conditions, significantly altered the community properties.

### 2.5. Pronounced Differences Exist in Microbial Metabolic Capacity Across the Concentration Gradient

**Figure 5:**
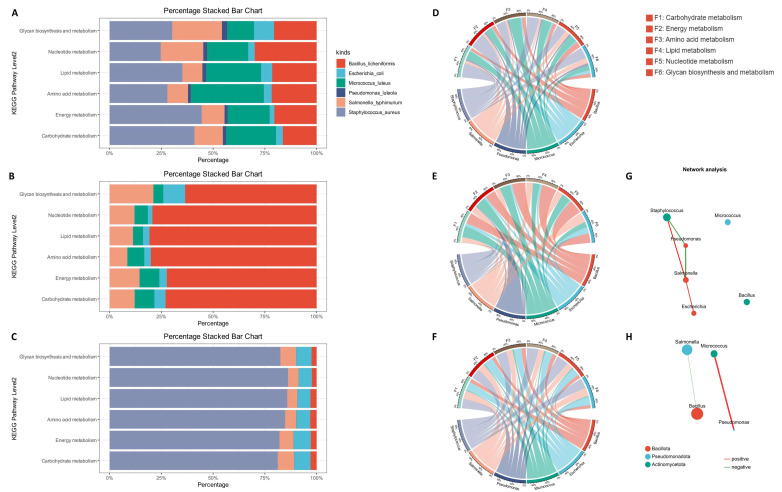
Analysis of species and metabolic function contributions. (**A**–**C**) Stacked chart of species metabolic levels, using TPM as the measure of expression levels. (**A**) pre. (**B**) post 0.3. (**C**) post 1.0. (**D**–**F**) Chord diagram of species metabolic capability, using TPM as the expression level metric, with TPM correction of each species after NR annotation of metagenomic data and performing normalization for different species and metabolic functions. (**D**) pre. (**E**) post 0.3. (**F**) post 1.0. (**G**,**H**) Single-factor network analysis based on KEGG Pathway Level 1 Metabolism. (**G**) post 1.0 (**H**) post 0.3.

To further explore the shaping mechanisms underlying compositional differences in the synthetic community under different nutritional conditions, we mined the metabolic capacity of the community at the transcriptional level based on KEGG annotation. We constructed a gene set from the KEGG-annotated metatranscriptomic data and selected six important metabolic domains for investigation: energy metabolism, amino acid metabolism, lipid metabolism, carbohydrate metabolism, nucleotide metabolism, glycan biosynthesis and metabolism. We generated an NR annotation table for this gene set to explore the relationship between species and metabolic functions. In the association analysis between the relative abundances of species and functions (Figure 5A–C), we found that in the pre-cultivation group, *Pseudomonas luteola* and *Escherichia coli* had lower metabolic levels, whereas the other four bacteria had similar metabolic levels. In the post-cultivation 0.3 group and 1.0 group, as expected, *Bacillus licheniformis* and *Staphylococcus aureus*, which occupied dominant positions in the community, had the highest metabolic levels and occupied an absolute dominant position in the functional composition of the community.

Considering the significant differences in the numbers of different species, we corrected transcript abundance using the species proportions obtained from NR annotation, in order to measure the metabolic capacity of different microorganisms per unit number, and plotted chord diagrams (Figure 5D–F). By analyzing these data, we found that in the pre-cultivation group, *Staphylococcus aureus* and *Micrococcus luteus* had the strongest metabolic capacity. In the 0.3 group, *Bacillus licheniformis* and *Micrococcus luteus* had the strongest metabolic capacity. *Salmonella typhimurium* was next, whereas the metabolic capacity of *Staphylococcus aureus* decreased greatly. In the 1.0 group, the microorganism with the strongest metabolic capacity was still *Staphylococcus aureus*, followed by *Escherichia coli* and *Salmonella typhimurium*, and the metabolic capacity of *Micrococcus luteus* decreased. Interestingly, the advantage of *Staphylococcus aureus* in metabolic capacity in the 1.0 group was not significant, showing little difference from that before cultivation, but its metabolic capacity decreased significantly in the 0.3 group. In addition, the metabolic capacity of *Bacillus licheniformis* in the 0.3 group increased compared with that before cultivation, but the difference between the 1.0 group and pre-cultivation was not obvious.

Subsequently, we investigated the interspecies interactions within the microbial community. Single-factor network analysis was employed to analyze the metabolic correlations between species in the samples (Figure 5G,H). The results showed that under nutrient-sufficient conditions, the interspecies metabolic interactions were more complex. *Salmonella typhimurium* exhibited positive correlations with both *Staphylococcus aureus* and *Escherichia coli*, whereas *Pseudomonas luteola* was negatively correlated with *Staphylococcus aureus* and *Salmonella typhimurium*. Under nutrient-limited conditions, however, the metabolic activities of the species appeared to be more isolated. Only a positive correlation between *Micrococcus luteus* and *Pseudomonas luteola* and a weak negative correlation between *Salmonella typhimurium* and *Bacillus licheniformis* were observed. By distinguishing microorganisms belonging to different phyla with differently colored circles, we found that microorganisms tended to interact metabolically with distantly related species.

Collectively, at the metabolic level, *Staphylococcus aureus* is more adapted to higher nutritional conditions, while *Bacillus licheniformis* is more suitable for lower nutritional conditions. Moreover, under competitive disadvantage, *Bacillus licheniformis* has stronger adaptability. In addition, we can find that in the 1.0 group, the metabolic capacity of *Salmonella typhimurium* and *Escherichia coli* increased, marking that high nutritional conditions are overall more suitable for the survival of conditionally pathogenic bacteria in the synthetic community. Meanwhile, single-factor network analysis suggested that the metabolic interactions among opportunistic pathogens were more complex under nutrient-rich conditions.

### 2.6. Striking Differences Exist in the Composition of Virulence Factors in Synthetic Microbial Communities Under Different Concentrations

**Figure 6:**
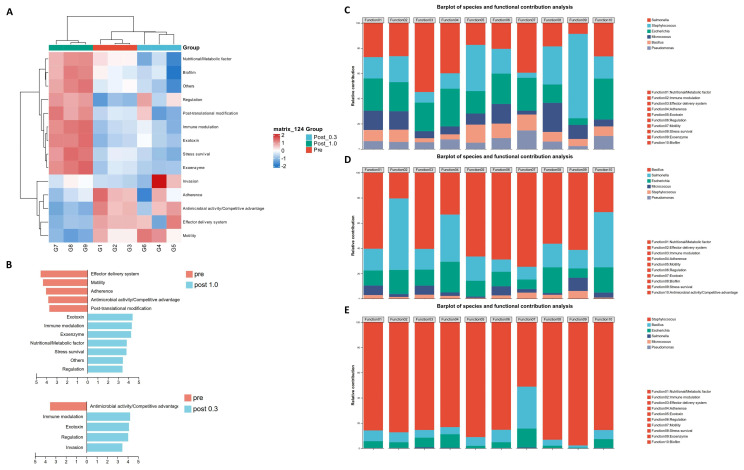
Analysis of virulence factors in synthetic communities. (**A**) Heatmap of VF Category. (**B**) Analysis of LEfSe. Using Reads Number as abundance index, Wilcoxon rank-sum test. (**C**–**E**) Stacked chart of species and functional contributions, using TPM as the abundance metric. (**C**) pre. (**D**) post 0.3. (**E**) post 1.0.

Finally, we studied the potential pathogenicity of artificial communities. We performed VFDB virulence factor annotation on the metagenomic data to explore the potential pathogenicity of different communities. The heatmap displays the abundance indicators of virulence factors under different nutritional conditions (Figure 6A). Most virulence factors (VFs) categories showed upregulated expression in the post 1.0 group, while in the post 0.3 group, expression was downregulated, unchanged, or slightly upregulated. Only a few factors, such as motility, invasion, adhesion, and effector delivery systems, showed significant downregulation in the post 1.0 group, with expression either unchanged or upregulated in the post 0.3 group. It can be seen that, compared to the high-nutrient community dominated by *Staphylococcus aureus*, the community dominated by *Bacillus licheniformis* under lower nutrient conditions had relatively weaker virulence. Combining Linear discriminant analysis Effect Size analysis (LEfSe), we identified characteristic virulence factor types in each group (Figure 6B). For the post 1.0 group, the greatly upregulated virulence factor types included immune modulation, exotoxins, exoenzymes, nutrient metabolism factors, survival stress, and regulation. For the post 0.3 group, characteristic virulence factor types included invasion, exotoxins, regulation, and immune modulation. This shows that the virulence factor profiles of the microbiota differ across different nutrient concentration gradients, and the infection intensity and symptoms may also vary.

To further investigate the primary pathogenic bacteria under different concentration gradients, we annotated species for the top ten virulence factor types by abundance at each concentration and studied the functional contributions of different species. The stacked bar charts quantify the virulence of different species in the pre-cultivation, post 0.3, and post 1.0 groups (Figure 6C–E). It can be observed that in the pre-cultivation community, the virulence of each species was relatively balanced, with *Salmonella* and *Staphylococcus* showing relatively higher virulence. After cultivation, the phenomena in both the post 0.3 and post 1.0 groups were as expected, with *Bacillus licheniformis* and *Staphylococcus aureus* showing the highest virulence. Interestingly, in the post 0.3 group, *Salmonella typhimurium* performed above its proportional representation in several indicators. Given that *Bacillus licheniformis* infections are relatively rare, *Salmonella typhimurium* is likely the most dangerous potential pathogen in the post 0.3 group. This suggests that under low-nutrient conditions, *Bacillus licheniformis* has strong antagonistic activity against *Staphylococcus aureus*, but its antagonism against *Salmonella typhimurium* is limited. This may be due to *Salmonella typhimurium*, as a Gram-negative bacterium, being less sensitive to certain antimicrobial peptides and having more virulence factors related to antimicrobial activity and competitive activities.

## 3. Discussion

**Figure 7:**
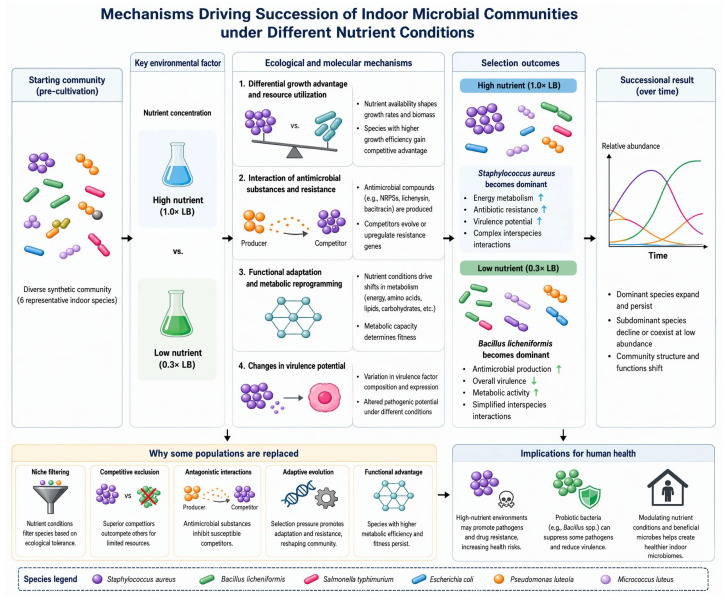
Successional direction and mechanisms of synthetic microbial communities. When the artificial indoor microbial community is placed under different nutrient conditions, it undergoes succession in divergent directions driven by the combined action of multiple competitive and antagonistic mechanisms. After succession, the communities exhibit distinct characteristics at multiple scales. Upward arrows represent pathway upregulation, while downward arrows represent pathway downregulation. Created in Tapnow (https://www.tapnow.ai/ (accessed on 4 May 2026)).

By constructing artificial microbial communities derived from indoor sources with metagenomic and metatranscriptomic approaches, we systematically investigated the effects of different nutritional conditions on the direction of microbial community succession, species competition mechanisms, and their relationship with human health. The results showed that nutrient concentration significantly affected the species composition and functional characteristics of the communities: under nutrient-rich conditions (1.0× LB medium), *Staphylococcus aureus* was the dominant species, and the community showed higher energy metabolism, antibiotic resistance, and virulence potential. While under low-nutrient conditions (0.3× LB medium), *Bacillus licheniformis* took the leading role. The community metabolism was active, but its pathogenicity was relatively low. These findings reveal that nutrient conditions acting as a key environmental factor can drive indoor microbial communities to evolve in different directions by regulating competition among microbes (Figure 7).

More and more studies indicate that interactions within bacterial communities can affect the resistance of strains to antibiotics [30,31]. In addition, environmental antibiotics can also change the composition, adaptive phenotypes, and genotypes of microbial community members [32]. In this study, we found that *Bacillus licheniformis* significantly upregulated the expression of various antimicrobial substances (such as NRPSs) under low-nutrient conditions. This phenomenon may explain its competitive advantage against opportunistic pathogens such as *Staphylococcus aureus* and *Salmonella typhimurium*. However, *Staphylococcus aureus* upregulated multiple antimicrobial peptide resistance genes under nutrient-rich conditions, enhancing its tolerance to antimicrobial active substances secreted by *Bacillus licheniformis*. Considering that *Staphylococcus aureus* exhibits relatively high potential resistance to natural antimicrobial substances under different environmental conditions, this dynamic game between “antimicrobial substances-resistance genes” may be one of the core mechanisms determining the succession direction of the community [33,34].

At the metabolic function level, analysis of metabolic capacity per unit abundance showed that *Staphylococcus aureus* exhibited stronger metabolic capacity under nutrient-rich conditions, whereas *Bacillus licheniformis* performed better metabolically under low-nutrient conditions. The results indicated a divergence in their adaptation to nutrient conditions. In addition, in the nutrient-rich group, the metabolic capacity of opportunistic pathogens (such as *Salmonella typhimurium* and *Escherichia coli*) was also significantly enhanced, suggesting that high-nutrient environments may synergistically promote the survival of multiple potential pathogens and increase health risks in indoor environments. This conclusion has been strongly supported by field studies [35]. Virulence factor analysis further supported this conclusion. In the nutrient-rich group, the community dominated by *Staphylococcus aureus* had multiple virulence-related genes, including immune regulation, exotoxins, and nutrient metabolism factors, showing stronger pathogenic potential. In contrast, in the low-nutrient group, although *Bacillus licheniformis* was dominant, *Salmonella typhimurium* still showed a prominent contribution in virulence factors. The stealthy transmission and high pathogenicity of *Salmonella* have already been elucidated [36]. It means that it may still pose a potential threat under low-nutrient conditions.

Beyond the dominant competitors, our results also revealed the fate of opportunistic pathogens that persist as minor but medically significant populations. Although Staphylococcus aureus and Bacillus licheniformis dominated the communities under nutrient-rich and nutrient-limited conditions, respectively, we observed that nutritionally favorable environments also allowed the persistence or metabolic enhancement of subdominant opportunistic pathogens such as Salmonella typhimurium and Escherichia coli. In the 1.0× LB group, the increased metabolic activity of these two strains and their greater contribution to virulence factors suggest that high-nutrient conditions may inadvertently create niches that accommodate multiple potential pathogens at the same time, even if they are competitively inferior to the dominant species [37]. In the low-nutrient group, although the overall pathogenicity of the community declined, Salmonella typhimurium still retained a high contribution in virulence-associated categories such as invasion and immune modulation. This phenomenon may reflect the intrinsic recalcitrance of Gram-negative pathogens: their outer membrane architecture confers reduced susceptibility to cationic antimicrobial peptides, and their extensive regulatory networks enable rapid exploitation of transient nutrient pulses or spatially heterogeneous microenvironments [38]. Therefore, this finding further emphasizes that risk assessment of indoor microbiomes cannot be confined to the dominant or most abundant species and highlights the importance of monitoring how environmental interventions inadvertently affect low-abundance but high-risk opportunistic pathogens [39].

A major research focus of the indoor microbiome is to use probiotic microorganisms to reduce multidrug-resistant organisms, which are acquired in hospitals and households [40]. *Bacillus* is a class of traditional probiotics [41], and it has long been used in the fields of medicine, the food industry, and ecosystem restoration [42,43]. Given that *Bacillus* are common, generally harmless, and capable of producing a variety of antimicrobial compounds, researchers have attempted to introduce *Bacillus* or its bioproducts onto surfaces prone to microbial growth in order to reduce the pathogenicity and resistance of microbiomes [44,45]. Our study confirmed the antagonistic effect of *Bacillus licheniformis* against multiple pathogenic bacteria, especially showing its antagonistic ability against *Staphylococcus aureus* under low-nutrient conditions. This conclusion is consistent with findings from studies in the gut [46]. *Bacillus licheniformis* gains a competitive advantage in low-nutrient environments by secreting antimicrobial active substances. It is worth further exploring that this antagonistic effect exhibits a certain degree of selectivity. *Bacillus licheniformis* shows significant inhibitory effects on *Staphylococcus aureus*, which is also a Gram-positive bacterium, but its inhibitory effect on Gram-negative bacteria such as *Salmonella typhimurium* is relatively limited, which may be related to the natural barrier effect of the outer membrane structure of Gram-negative bacteria against certain antimicrobial peptides [47,48]. This finding suggests that, in real indoor environments, introducing or enriching probiotic *Bacillus licheniformis* may inhibit some opportunistic pathogens and then regulate the indoor microbiome to evolve in a healthier direction.

Indoor microbiome research is at the forefront of environmental science. However, current studies mainly focus on in situ studies and sampling studies of microorganisms in indoor environments [49], and have not established a stable and reproducible laboratory model. Our work innovatively introduced a synthetic microbial community model into indoor microbiome research and systematically analyzed the driving mechanisms of community succession under different nutrient conditions through multi-omics approaches. Compared with traditional studies based on natural sampling, our model system is controllable and highly reproducible, removes uncontrollable variables, and can more clearly reveal microbial interactions and functional evolution. Moreover, with the proposal and development of the concept of “healthy buildings” in recent years, our study also provides a screening model for selecting beneficial indoor microbial strains and creating a healthier living environment [50,51].

However, our research also has certain limitations. First, the synthetic community only includes six representative strains. Therefore, it cannot fully simulate the complexity and diversity of real indoor microbial communities. Second, the cultivation condition is a liquid homogeneous system, and this does not consider real factors such as surface attachment, spatial heterogeneity, and desiccation stress. In terms of simulating natural environments, some studies can already serve as references [52,53]. Future studies can further introduce systems with multiple species, multiple substrates, and multiple environmental factors, combined with temporal dynamic analysis and functional validation experiments so that the evolutionary patterns and health effects of indoor microbial communities will be further revealed.

Additionally, further research should give priority to enhancing the ecological realism of synthetic indoor models. First, expanding the species pool to include phylogenetically diverse bacteria as well as fungi would more comprehensively capture the full taxonomic spectrum of real indoor microbiomes. Second, introducing surface-attached growth systems—for example, multi-surface microcosms incorporating common building materials such as stainless steel and drywall—would more faithfully simulate the stresses encountered on indoor surfaces. Third, adopting longitudinal sampling with high temporal resolution, rather than solely endpoint comparisons, would enable a better investigation of the successional dynamics of microbial communities. Finally, bridging laboratory models with field validation is essential: placing potential probiotics screened in the laboratory into real indoor environments would allow effective evaluation of their translational medical value. Such integrated research approaches will not only deepen our mechanistic understanding of indoor microbial ecology but also provide actionable strategies for microbiome-informed building design and public health interventions.

In summary, the study reveals that nutrient conditions significantly influence the succession direction and functional characteristics of indoor microbial communities by regulating the game between antimicrobial substances and resistance. The results provide a new perspective for understanding the ecological evolution mechanisms of indoor microbiomes. Furthermore, the results provide a theoretical basis for future interventions to regulate indoor microbial composition and reduce health risks through environmental control.

## 4. Materials and Methods

### 4.1. Experimental Reagents and Strains

The main reagent used in the experiment, Luria–Bertani (LB) medium (batch number: 20240514), was purchased from Qingdao Hope Biotechnology Co., Ltd. (Qingdao, China).

All the strains used in this study were obtained from the China Center for Type Culture Collection (CCTCC) (Wuhan, China), including six strains: *Bacillus licheniformis* (CCTCC AB 91061), *Staphylococcus aureus* (CCTCC AB 91093), *Salmonella typhimurium* (CCTCC AB 2014174), *Pseudomonas luteola* (CCTCC S 2014034), *Escherichia coli* (CCTCC AB 2017070), and *Micrococcus luteus* (CCTCC AB 2017026).

### 4.2. Bacterial Growth Curve Measurement

The six bacterial suspensions to be tested were first activated and then inoculated into 1.0× LB and 0.3× LB media once the optical density at 600 nm (OD600) reached 0.9. For each prepared medium, 200 μL of bacterial suspension was added and mixed thoroughly, with three replicates per group. After mixing, 100 μL of the inoculated medium was transferred into a 96-well plate, and the OD600 was measured using a microplate reader (BioTek, Winooski, VT, USA). Blank 1.0× LB and 0.3× LB media served as controls, and the initial values at 0 h were recorded. The tubes were then incubated in a shaker at 28 °C and 150 rpm, and the OD600 was measured every two hours for a total duration of 20 h using the same method. The recorded OD values were used to plot growth curves.

### 4.3. Strain Expansion and Mixed Cultivation

LB medium was prepared in 0.3× and 1.0× LB solutions, each dispensed into three conical flasks with a volume of 200 mL. The flasks were sealed and sterilized by autoclaving. After determining the bacterial concentration, each strain was expanded in LB medium. Six bacterial strains, previously inoculated and activated in LB medium, were taken, and 200 μL of each was transferred into a 96-well plate. The absorbance at 600 nm was measured using a microplate reader (BioTek, Winooski, VT, USA). Upon reaching an OD600 of 0.9, equal amounts of bacterial suspension were inoculated into 1.0× LB and 0.3× LB media and assigned to two groups: an uncultured control and a co-culture group maintained for two months, with three replicates per group.

### 4.4. De Novo Genome Sequencing

Complete bacterial genome maps were obtained using a combination of second-generation (Illumina) and third-generation (single-molecule PacBio) sequencing technologies. For each sample, no less than 10× PacBio sequencing data and 100× Illumina sequencing data of the genome were obtained simultaneously.

Genomic DNA was collected and purified, fragmented using a Covaris instrument (Covaris, Woburn, MA, USA), and used to construct a genomic sequencing library. A and B adapters were ligated, self-ligated fragments were removed, and the resulting fragments were size-selected by agarose gel electrophoresis. The library was denatured with sodium hydroxide to generate single-stranded DNA fragments, and bridge PCR was performed to amplify the DNA molecules prior to Illumina sequencing.

The concentration of genomic DNA was measured using TBS380 and Nanodrop 2500 instruments (Thermo Fisher Scientific, Waltham, MA, USA) to ensure sufficient DNA quality for subsequent experiments. The criteria applied were that the genomic DNA should be intact, with an OD260/280 ratio of 1.8–2.0 and a total amount of no less than 10 μg. The genome was then fragmented using the G-tube method for single-molecule library construction, followed by single-molecule PacBio sequencing.

After sequencing, quality control was performed on the obtained data. Unicycler (version 0.4.8) was used for third-generation sequence assembly, and Pilon (version 1.24) was applied for sequence correction. Prodigal (version 2.6.3) was used to predict chromosomal genes, and GeneMarkS (version 4.3) was used for plasmid gene prediction.

### 4.5. Metagenomic Sequencing

Samples were collected from the uncultured group and the two-month mixed culture group. Genomic DNA was extracted and analyzed by 1% agarose gel electrophoresis. The DNA was fragmented to approximately 350 bp using a Covaris M220 instrument (Covaris, Woburn, MA, USA). Paired-end libraries were constructed by ligating specific adapters to both ends of the DNA fragments, followed by magnetic bead selection and removal of self-ligated adapter fragments. The library templates were enriched by PCR amplification, and single-stranded DNA fragments were generated by sodium hydroxide denaturation. Bridge PCR was then performed to amplify DNA molecules, ensuring efficiency and accuracy during sequencing. Illumina sequencing was carried out as required, with template DNA fragment sequences determined by collecting fluorescent signals in each cycle. During data analysis, raw reads were first trimmed of low-quality regions, and adapter contamination was removed using Trimmomatic (version 0.39). Sequencing data were assembled using MEGAHIT (version 1.2.9), and gene prediction was performed on the assembled contigs using Prodigal. Sequence clustering and alignment were performed using CD-HIT (version 4.6.1) and SOAPaligner (version 2.21), and quality control was conducted with Fastp (version 0.23.0).

### 4.6. Metatranscriptome Sequencing

Samples from the uncultured and two-month mixed culture groups were collected and treated with an ion fragmentation reagent and random primers to fragment the target RNA and allow the primers to complement the RNA. Reverse transcription reagents and second-strand synthesis reagents were added to synthesize double-stranded cDNA. The cDNA products were end-repaired, A-tailed, and ligated to adapters; magnetic beads were used to remove adapter self-ligation fragments. The library templates were enriched by PCR amplification and denatured with sodium hydroxide to generate single-stranded DNA fragments. Bridge PCR was performed to amplify DNA molecules, followed by Illumina sequencing. Quality control of the original sequencing data was conducted by trimming low-quality reads and reads containing Ns. Assembly was performed using Trinity, after which rRNA sequences were removed using SortMeRNA (version 4.3.6), and gene prediction was carried out with Prodigal. Sequence alignment was then performed using CD-HIT, gene abundance was calculated using RSEM (version 1.3.3), and final quality control was conducted with Fastp.

### 4.7. Sequencing Data Annotation

Sequence data were annotated and analyzed based on multiple databases. Non-Redundant Protein Sequence Database (NRDB) was used for species annotation. The non-redundant gene set was compared with the NR database using DIAMOND (version 2.0.13) software (http://ab.inf.uni-tuebingen.de/software/diamond/ (accessed on 16 July 2025)), and species annotation was obtained through the taxonomy information database corresponding to the NR database [54]. Kyoto Encyclopedia of Genes and Genomes (KEGG) database (http://www.kegg.jp/kegg/kegg1.html (accessed on 20 October 2025)) was used for KEGG functional annotation [55,56]. Virulence Factors of Bacterial Pathogens (VFDB) (https://www.mgc.ac.cn/VFs/ (accessed on 20 October 2025)) was used for predicting virulence genes [57].

### 4.8. Data Analysis and Visualization Processing

The data were analyzed on the online tool of Hiplot ORG (https://hiplot.org (accessed on 12 November 2025)) and Majorbio Cloud Platform (https://www.majorbio.com/tools (accessed on 12 November 2025)) [58,59].

## 5. Conclusions

In this study, we constructed a synthetic indoor microbial community and, through integrated metagenomic and metatranscriptomic analyses, systematically investigated the effects of different nutrient conditions on community succession, functional evolution, and pathogenic potential. Our results demonstrate that nutrient availability serves as a key environmental filter that reshapes community structure by modulating the antagonistic interplay between antimicrobial compounds and resistance mechanisms. Under nutrient-rich conditions, *Staphylococcus aureus* dominated, exhibiting enhanced energy metabolism, antimicrobial resistance, and virulence potential. In contrast, under nutrient-limited conditions, *Bacillus licheniformis* outcompeted other species through the upregulation of antimicrobial biosynthetic pathways, leading to a community with lower overall pathogenicity. Notably, the competitive advantage of *Bacillus licheniformis* was partially selective, showing stronger suppression against Gram-positive pathogens than against Gram-negative bacteria such as *Salmonella typhimurium*. These findings highlight the potential of leveraging probiotic *Bacillus* species to steer indoor microbiomes toward healthier states, while also underscoring the need for comprehensive risk assessments beyond dominant species. By introducing a reproducible synthetic community model combined with multi-omics approaches, this work provides a controlled experimental framework for deciphering ecological mechanisms in indoor environments and offers theoretical foundations for microbiome-based interventions aimed at improving public health in urban settings. In the future, building on the findings of this study, we will refine and expand the synthetic indoor microbial community model and continue to explore probiotics with the potential to improve the indoor ecological microenvironment.

## Figures and Tables

**Table 1:** Strains for Creating Synthetic Microbial Communities.

Scientific Name and Strain Identification Number	Indoor Distribution	Physiological Characteristic	Relationship with Human Health
*Bacillus licheniformis* (CCTCC AB 91061)	The majority of indoor areas	Gram-positive bacteria, can form spores, facultative anaerobic	Probiotics, Can antagonize pathogenic bacteria, disrupt biofilm
*Staphylococcus aureus* (CCTCC AB 91093)	Surfaces of the skin, hospitals, bathrooms, and restrooms	Gram-positive bacteria, facultative anaerobic, strong drug resistance	Opportunistic pathogens, likely to cause infections in the skin, tissues, and organs
*Salmonella typhimurium* (CCTCC AB 2014174)	Hospital, restroom	Gram-negative bacillus, facultative anaerobic, sensitive to heat	Invasive pathogenic bacteria containing multiple endotoxins, highly virulent
*Pseudomonas luteola* (CCTCC S2014034)	Soil and waterlogged areas	Gram-negative bacillus, aerobes, strong drug resistance	Opportunistic pathogens, sometimes leading to serious infections
*Escherichia coli* (CCTCC AB 2017070)	Surfaces of the skin, bathrooms	Gram-negative bacillus, facultative anaerobic	Opportunistic pathogens, overbreeding can lead to infection
*Micrococcus luteus* (CCTCC AB 2017026)	kitchen	Gram-positive bacteria, aerobes, mesophilic bacteria	rarely causing infections, with only a few cases reported.

**Table 2:** The Genome Attribute CCTCC AB 91061 and CCTCC AB 91093.

Attribute	CCTCC A91061	CCTCC A91093
Genome size (bp)	4,357,622	2,786,253
No. of contigs	1	1
depth	76.91	249.58
% GC content	45.87	32.88
Protein coding genes	4523	2559
tRNA encoding genes	81	56
rRNA encoding genes	24	19

## Data Availability

All sequencing data supporting this study have been deposited in the National Center for Biotechnology Information (NCBI) under the primary accession code PRJNA1441380. The other data that support the findings of this study are available from the corresponding author, Fang Peng, upon reasonable request.

## Supplementary Materials

The following supporting information can be downloaded at: https://www.mdpi.com/article/10.3390/ijms27104238/s1.

## Abbreviations

The following abbreviations are used in this manuscript:

AMP	Antimicrobial peptide
CCTCC	China Center for Type Culture Collection
FC	Fold change
KEGG	Kyoto Encyclopedia of Genes and Genomes
LB	Luria–Bertani medium
NRDB	Non-Redundant Protein Sequence Database
NRPSs	Nonribosomal peptide synthetases
OD	Optical density
PE	Paired-end
PCA	Principal Component Analysis
TPM	Transcripts per million
VFDB	Virulence Factors of Bacterial Pathogens
