# Supplementary material for: Comparative Metagenomic Studies Reveal Different Evolutionary Directions of Synthetic Indoor Microbial Communities Under Different Nutritional Conditions
Source: Int J Mol Sci. 2026 May 10;27(10):4238. doi: 10.3390/ijms27104238 (PMC13207052; doi:10.3390/ijms27104238)
Supplement: Supplementary file 1 [file ijms-27-04238-s001.zip › ijms-4276898-supplementary.pdf]

Table S1. Antimicrobial substances annotated from the genome of *Bacillus licheniformis*

Gene ID	Gene len (bp)	Protein len (aa)	Description in NRDB
Gene 0504	10749	3582	lichenysin non-ribosomal peptide synthetase *LicA*
Gene 0505	10767	3588	non-ribosomal peptide synthetase *LicB*
Gene 0506	3849	1282	MULTISPECIES: lichenysin non-ribosomal peptide synthetase *LicC*
Gene 2887	297	98	MULTISPECIES: antimicrobial peptide LCI
Gene 4170	7158	2385	non-ribosomal peptide synthetase *dhbF*
Gene 4346	1509	502	MULTISPECIES: D-alanine--poly(phosphoribitol) ligase subunit *DltA*
Gene 4573	381	126	MULTISPECIES: lactococcin 972 family bacteriocin
